# Visual illusions in Parkinson's disease: an interview survey of symptomatology

**DOI:** 10.1111/psyg.12771

**Published:** 2021-10-06

**Authors:** Chinami Sasaki, Kayoko Yokoi, Hiroto Takahashi, Tomoyuki Hatakeyama, Koji Obara, Chizu Wada, Kazumi Hirayama

**Affiliations:** ^1^ Yamagata Prefectural University of Health Sciences Yamagata Japan; ^2^ National Hospital Organization Akita Hospital Akita Japan; ^3^ National Hospital Organization Miyagi Hospital Yamamoto Japan

**Keywords:** optical illusions, Parkinson's disease, vision disorders, visual perception

## Abstract

**Background:**

Several types of visual illusions can occur in Parkinson's disease (PD). However, the prevalence and types of specific illusions experienced by patients with PD remain unclear. This study aimed to investigate the types of illusions.

**Methods:**

A questionnaire of visual illusions was developed through a literature review in consultation with clinicians and neurologists. Based on the questionnaire, 40 consecutive patients with PD were asked a series of Yes/No questions regarding 20 types of visual illusions since the onset of PD. If participants answered ‘Yes’, they were then asked to detail their experience(s).

**Results:**

In total, 30 patients with PD had experienced visual illusions since disease onset; among them, 25 were still experiencing them at the time of the study. The most commonly observed illusion types were dysmorphopsia, complex visual illusions, metachromatopsia, and diplopia. Other observed illusions included textural illusions, macropsia, micropsia, teleopsia, pelopsia, kinetopsia, akinetopsia, Zeitraffer/Zeitlupen phenomena, tilt illusion, upside‐down illusion, and palinopsia. Additionally, aberrant perception of surface orientation (inclination) was reported, which is yet to be reported in association with any disease. Visual illusions had detrimental effects on the patients’ daily lives in some cases.

**Conclusions:**

Systematic interviews regarding the incidence and details of visual illusions experienced by patients with PD could offer important information regarding their quality of life.

## INTRODUCTION

The currently accepted National Institute of Neurological Disorders and Stroke–National Institute of Mental Health diagnostic criteria emphasise minor hallucinations as the most common psychotic symptom in Parkinson's disease (PD).^1^ Minor hallucinations comprise three types of hallucinatory experiences: presence hallucinations (or feeling of presence), passage hallucinations, and visual illusions. These visual illusions may denote the complex phenomenon of one object being perceived as another kind of object (e.g. a branch being seen as a cat). Moreover, there are simple visual illusions, where just one of the features, including colour, shape, size, distance, motion, tilt, number, or the temporal aspect, is altered. Both complex and simple visual illusions may occur in PD.^2^, ^3^, ^4^ Reported simple illusions in patients with PD include kinetopsia, dysmorphopsia, metachromatopsia, macro‐/micropsia, tele‐/pelopsia,^2^ selective diplopia,^3^ and tilt illusions.^4^ Further, other types of illusions have been reported after localised brain injury and in association with migraines and epileptic seizures. However, there has been no study on the illusion types that can occur in PD; moreover, the only studies that have reported specific details regarding simple illusions in patients with PD have exclusively focused on selective diplopia^3^ or tilt illusions.^4^

This study aimed to investigate the types, prevalence, and details of visual illusions experienced by patients with PD through an interview survey.

## METHODS

### Participants

We included 40 consecutive patients (outpatients or inpatients) with PD from the National Hospital Organization Akita National Hospital. PD was diagnosed based on the United Kingdom Parkinson's Disease Society Brain Bank criteria. We excluded patients with a history of central nervous system illness (i.e. stroke, migraine, epilepsy, etc.) or psychiatric illness, evidence of non‐PD‐related abnormalities on cranial magnetic resonance imaging (MRI), hearing loss, or binocular corrected near visual acuity (NVA) < 0.5. Patients who developed dementia within 1 year after being diagnosed with PD were also excluded based on the past medical records by neurologists. Table 1 summarises the demographic characteristics of the patients.

**Table 1:** Demographic, clinical, and neuropsychological features of PD patients with versus without visual illusions

	PD total (*n* = 40), mean (SD), [range]	PD with visual illusions (*n* = 30), mean (SD), [range]	PD without visual illusions (*n* = 10), mean (SD), [range]	*P*‐values
Age, years	64.4 (5.0), [56–78]	65.0 (4.8), [56–78]	62.4 (5.4), [56–72]	0.140
Sex, men/women	21/19	17/13	4/6	0.473
Education, years	14.1 (2.1), [12–18]	13.7 (2.1), [12–18]	15.2 (1.7), [12–16]	0.089
Disease duration, years	7.0 (3.6), [1–15]	7.7 (3.4), [2–15]	5.0 (3.3), [1–11]	0.036*
Hoehn & Yahr stage	2.5 (0.7), [1–4]	2.6 (0.7), [1–4]	2.2 (0.4), [2–3]	0.067
MDS‐UPDRS Part III	45.4 (20.9), [9–80]	49.3 (21.8), [9–80]	33.4 (12.4), [20–59]	0.024*
Levodopa equivalent dose, mg	344.8 (176.6), [133.0–865.5]	371.0 (191.3), [133.0–865.5]	266.3 (90.4), [133.0–424.0]	0.177
CUCVT (Max: 10)	9.1 (1.0), [6–10]	8.9 (1.0), [6–10]	9.6 (0.7), [8–10]	0.067
MoCA‐J (Max: 30)	26.3 (3.1), [19–30]	25.5 (3.0), [19–30]	28.8 (2.0), [24–30]	0.001**
RAVLT (Max: 15)	10.2 (1.7), [6–13]	9.7 (1.5), [6–13]	11.5 (1.4), [8–13]	0.001**
Overlapping figure (Max: 6)	1.0 (1.3), [0–5]	1.3 (1.4), [0–5]	0.2 (0.4), [0–1]	0.018*
Noise Pareidolia, %	10.0 (13.0), [0–50]	12.8 (13.8), [0–50]	1.5 (3.9), [0–12.5]	0.004**

Significant at *P < 0.05 and **P < 0.01. Fisher's exact test and the Mann–Whitney *U*‐test were used for categorical and continuous variables, respectively. All *P*‐values are two tailed. PD, Parkinson's disease; SD, standard deviation; MDS‐UPDRS Part III, Movement Disorder Society‐Unified Parkinson's Disease Rating Scale Part III; CUCVT, City University Colour Vision Test; MoCA‐J, Japanese version of the Montreal Cognitive Assessment; RAVLT, Ray Auditory Verbal Learning Test.

All participants provided written informed consent after receiving a detailed description of the study. This study was approved by the ethics committee of the National Hospital Organization Akita National Hospital and conducted in accordance with the Declaration of Helsinki.

### Background motor and neuropsychological assessments

Patients were evaluated in the ‘ON’ state using the Unified Parkinson's Disease Rating Scale (UPDRS) Part III. General cognitive function was evaluated based on the Japanese version of the Montreal Cognitive Assessment (MoCA‐J) score.^5^ Long‐term memory was evaluated according to the number of words recalled after a 30‐min break on the Ray Auditory Verbal Learning Test (RAVLT). Visuospatial perception was evaluated based on the score in the overlapping figure identification task of the Visual Perception Test for Agnosia (VPTA).^6^ Colour vision was evaluated based on the number of correct responses on the City University Colour Vision Test (CUCVT).^7^ Further, patients were evaluated based on the number of pareidolic responses confirmed on the Noise Pareidolia Test.^8^

### Questions regarding visual illusions

First, patients received detailed descriptions of the differences between hallucinations and (visual) illusions; moreover, they were informed that the interview questions would specifically regard the latter. The researcher regularly confirmed their understanding throughout the interview and assured them not to be concerned since visual illusions are occasionally experienced by patients with PD and are not aberrant signs. Subsequently, they were asked a series of Yes/No questions orally regarding unrealistic visual perceptions since PD onset (Table 2, section A). In case of affirmative responses, specific details regarding the experience were requested. During the patient's account, the researcher verified that the event referred to specific objects that really existed—e.g. when a patient said, ‘It looked like there were two of my TVs at home, side by side, on top of the TV stand’— to confirm it met the criteria for the visual illusion in question. Additionally, the patients were asked regarding the timing and frequency of each illusion. They were also asked whether they were still experiencing these illusions, and if so, when they began and stopped, as well as whether they had any difficulties in their daily life as a result of the occurrence of the type of visual illusion (Table 2, section B).

**Table 2:** Questions on visual illusions

A. The presence (or absence) of visual illusions
1. Has something that actually exists ever appeared to have a different colour from its actual colour? → metachromatopsia
2. Has the surface of something that actually exists seemed to appear different from its actual state? → textural illusion
3. Has a shape ever appeared to become distorted or deformed? → dysmorphopsia
4. Have things ever appeared to be bigger than what they actually were? → macropsia
5. Have things ever appeared to be smaller than what they actually were? → micropsia
6. Have things ever appeared to be farther away than where they actually were? → teleopsia
7. Have things ever appeared to be closer than where they actually were? → pelopsia
8. Have things that are not supposed to be moving ever appeared to be moving? → kinetopsia If yes, were the object's surroundings also moving? → not kinetopsia. Nystagmus etc. Or did only that object move and not its surroundings? → kinetopsia
9. Have things that are supposed to be moving ever appeared to be stationary? → akinetopsia
10. Has the movement of an item ever appeared to be faster than its actual speed? → Zeitraffer phenomenon
11. Has the movement of an item ever appeared to be slower than its actual speed? → Zeitlupen phenomenon
12. Have things ever appeared to be tilted or upside‐down as opposed to their actual direction? Please specify: Did they appear tilted? → tilt illusion Did they appear upside‐down? → upside‐down illusion
13. Has a single item ever appeared as though it were two or more items instead?
a. How many did they appear to be? Did you see two items? → cerebral diplopia or polyopia (two images) Did you see more? → polyopia (≥three images) If there were more than three items, approximately how many were there?
b. Did the items increase in number (after a while) when you were looking at them? → cerebral diplopia
c. Did they increase in number when you looked away from them? → polyopia
d. Did the item(s) only increase in number after (or while it was) moving? → polyopia
e. Did the item(s) increase in number only when you moved (or while you were moving)? → polyopia
14. Have you ever seen something, then seen it again despite it no longer being there?
a. Have you ever experienced seeing something once and then continued seeing the item despite the fact that it should no longer be there? → immediate perseveration
b. Have you ever experienced seeing something once and after a while, seen the item again despite the fact that it was no longer there? → palinopsia or hallucinatory palinopsia If the response is ‘Yes’, ask ‘After how much time did you see it’? Image recurred after several minutes →palinopsia Image recurred after several days to weeks →hallucinatory palinopsia
15. Has something that actually exists ever appeared to be something completely different? →complex visual illusion
B. Period and frequency of illusion occurrence, daily life‐related problems, etc.
1. How long has the optical illusion been occurring?
When does it begin and end?
Does it still persist?
2. How often does this optical illusion occur?
Please respond in the format of ‘a few times a day’ or ‘once a month’.
3. Have you experienced any difficulties in your daily life as a result of the occurrence of this type of visual illusion? What were the difficulties?
4. Is there anything you are concerned about regarding the optical illusions that we did not address?
5. If it is not too much to ask, could you draw an example of an optical illusion that you have experienced?

Questions were asked for each of the following types of visual illusion.

**Table:** 

Metachromatopsia:	Object colour appears different from that in reality^9^
Textural illusion:	Object surface appears different from that in reality
Dysmorphopsia:	Object shape appears distorted^10^
Macropsia:	Object appears larger than in reality^11^
Micropsia:	Object appears smaller than in reality^12^
Teleopsia:	Object appears more distant than in reality^13^
Pelopsia:	Object appears nearer than in reality^13^
Kinetopsia:	Stationary object appears to be moving^14^, ^15^
Akinetopsia:	Moving object appears to be stationary^16^
Zeitraffer phenomenon:	Motion of object appears faster than in reality^17^, ^18^
Zeitlupen phenomenon:	Motion of object appears slower than in reality^17^, ^19^
Tilt illusion:	Orientation of the visual scene appears tilted^20^
Upside‐down illusion:	Orientation of the visual scene appears inverted^20^

Additionally, *polyopia* and *cerebral diplopia* involve the perception of a single object as two or more objects.^21^ In polyopia, ≥two objects appear side by side because of movement—by the patients themselves, by their gaze, or by the original object. Polyopia can be divided into instances of seeing two images of the same object (‘polyopia (two images)’) and seeing three or more images of the same object (‘polyopia (≥three images)’). In contrast, in cerebral diplopia, the object ‘increases’ upon continuous viewing. Selective diplopia is a documented illusion in patients with PD, which is distinct from double vision caused by oculomotor dysfunction and is characterised by the doubling of a single object.^3^ This dysfunction applies to both polyopia (two images) and cerebral diplopia. *Visual perseveration* refers to an illusion category involving the continual perception of an object after it has left the visual field. Based on the timing of the illusory perception, it can be categorised into the following three forms: *immediate perseveration*, if the object is still apparent just after its disappearance; *palinopsia*, if it returns after a few minutes; and *hallucinatory palinopsia*, if it recurs after days or weeks.^21^ Further, the patients were asked about complex visual illusions.

We asked additional questions when it was necessary to distinguish visual illusions from other neurological symptoms and between similar types of illusions. When a patient confirmed experiencing akinetopsia, they were asked a supplementary question as to whether the scenery around the object also moved or not to confirm that the experience was not attributable to nystagmus. To differentiate between polyopia (two images) and cerebral diplopia, patients who provided positive responses were also asked supplementary questions on whether their gaze, body, or the focal object itself had moved before the apparent increase, as well as whether they had been previously staring at the object. To classify visual perseverations by type, supplementary questions were asked to determine the time which passed between seeing the original object and the illusion perception.

In Table 2 (section A), each question is followed by an arrow denoting the illusion/pathology—or pair requiring differentiation—recorded in the event of an affirmative (yes) response.

After going through the list of questions, the patients were asked if they had any other odd experiences besides the aforementioned specific illusions. Finally, they were provided coloured pencils and requested to draw a picture of what they had perceived, if possible (Table 2, section B).

### Questions regarding the visual hallucinations

We asked all the participants about the presence or absence of hallucinations since the onset of PD after explaining in detail the difference between visual illusion and visual hallucination, at a time more than 2 weeks later than the interview for visual illusions.

### Statistical analysis

Fisher's extract test and the Mann–Whitney *U*‐test were used to compare categorical and continuous variables, respectively, between the groups with and without visual illusions.

Since there was a need to ensure that the reported illusions were not confabulations due to other conditions that can affect patients with PD, including amnesia and frontal dysfunction, we validated the questionnaire's test–retest reliability. Consequently, the same questions were asked after 2 weeks to a randomly selected subset of participants (*n* = 6/40). This second interview was conducted by a different person blinded to the study details and the patients’ initial responses. Otherwise, the method of conducting the second interview was the same as the first. For all binary (Yes/No) questions in Table 2, Cohen's kappa coefficient (κ) was calculated to measure the degree of agreement between the patients’ first‐ and second‐round responses.

Statistical processing was performed using IBM SPSS Statistics version 22 for Windows (IBM Corp., Armonk, NY). Statistical significance was set at *P* < 0.05.

## RESULTS

### Background motor and neuropsychological assessments

In total, 30 patients reported at least one visual illusion, whereas 10 did not report any. Comparing the patients reporting versus those not reporting illusions, there were no significant between‐group differences in age, sex, education, Hoehn and Yahr stage, levodopa equivalent dose (LED),^22^ and colour vision on the CUCVT. None of the participants were taking anticholinergic drugs and only one patient was taking donepezil hydrochloride. The group with visual illusions had a longer disease duration, and worse scores for the UPDRS part III, the MoCA‐J assessment of general cognitive function, the RAVLT evaluation of long‐term memory, and the overlapping figure task of the VPTA measurement of visuospatial perception. A greater number of pareidolic responses on the Noise Pareidolia Test was also produced by the patients with visual illusions than by those without (Table 1).

### Reported illusions

**Figure 1:**
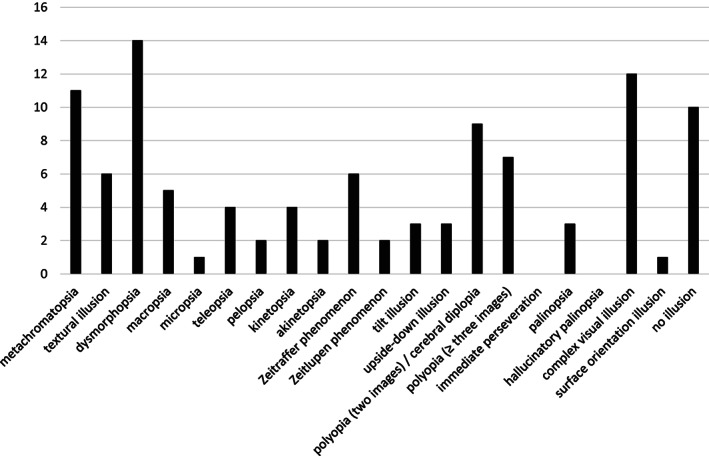
The number of patients reporting visual illusions according to type.

Figure 1 presents the number of patients who reported each illusion type surveyed in the questionnaire. Nearly every illusion was observed by at least one patient with the exceptions of immediate perseveration and hallucinatory palinopsia. Polyopia (two images) and cerebral diplopia were collectively counted as ‘polyopia (two images)/cerebral diplopia’ since none of the patients who provided positive responses could recall the situational details (i.e. motion of gaze, self, or object) prior to the start of the illusory increase. The most commonly experienced illusions were dysmorphopsia (*n* = 14), complex visual illusions (*n* = 12), metachromatopsia (*n* = 11), and polyopia (two images)/cerebral diplopia (*n* = 9). Some representative examples of different illusion types are presented in Table 3.

**Table 3:** Types of visual illusions and example of the patients’ experiences

Types of visual illusions	Examples of the patients’ experiences
Metachromatopsia	Son's blue car appears yellowish‐green or fluorescent green at times. Husband's dark‐blue jacket appeared pale sky blue when it was returned from the dry cleaner. Her husband denied that the colour had changed when she told him it had faded.
Textural illusion	The patient takes care not to trip while walking on a (flat) floor mat in hospital, which occasionally looked wavy and uneven. The pet guinea pig's fur looked stiff, like a hedgehog's [quills].
Dysmorphopsia	The hospital bed's shape appears bent at times, not straight, curving at the middle and distant points. A bicycle parked in front of the hospital appeared bent in two, at an angle of about 60°. Others’ faces look distorted at times.
Macropsia	Own smartphone appeared to be about as large as son's tablet. A bicycle at the hospital looked about 1.5 times larger than the surrounding ones, despite being the same model.
Micropsia	An apple on top of a table appeared to be the size of a cherry.
Teleopsia	The bed's legs occasionally appear far away. Starting 2 weeks ago, a utility pole near the patient's house occasionally appeared to be about 30 m distant.
Pelopsia	When driving, signboards and other surroundings occasionally appear closer than in reality. When going to the bathroom, the stair steps appear closer than in reality at times.
Kinetopsia	When putting a dog into its cage, the entire cage moves sideways at times. Moreover, the surrounding scene does not move in tandem. The wristwatch's rim appears to rotate at times. Blood vessels and other arm features sometimes appear to rotate in the opposite direction (of the rim) as well.
Akinetopsia	A bug, which others said was moving, appeared stationary to the patient. On the highway, a car driving in front of the patient seemed to suddenly stop, which caused him to change lanes; however, the car was still driving and was alongside him.
Zeitraffer phenomenon	A nursing assistant appeared to be walking at the speed of a bullet train. A clock's second hand suddenly and quickly made a complete revolution and returned to its original position.
Zeitlupen phenomenon	A clock's second hand appears to move more slowly at times. A ball hit by a student in a tennis court in front of the patient's house appeared to move slowly and appeared not to have arrived at the opponent when the latter swung the racket.
Tilt illusion	A doll at home appeared to be tilted to the left by about 45°. When a pet dog was digging a shallow hole, it appeared to do a ‘handstand’ on its front paws (by rotating 90°).
Upside‐down illusion	A utility pole in the neighbourhood appeared to be upside down, with the sky visible underneath. A friend's face appeared to be inverted.
Polyopia (two images)/cerebral diplopia	A television at home appeared to be two units, side by side, on top of the single TV stand. A normal medicine cup appeared as two cups, one atop the other, suspended in mid‐air.
Polyopia (≥three images)	A single (upright) pencil appeared as three pencils (lined up side by side). A soap bar in the washroom appeared as four bars (stacked on top of each other).
Palinopsia	An occupational therapist's face ‘re‐appeared’ for 3–4 min at 3–4 min after completion of the rehabilitation training. Chopsticks used for a meal ‘re‐appeared’ after 2 h.
Complex visual illusion	Wrinkles in sheets occasionally perceived as a human face. Felt grossed out by a table pattern, which was perceived as moving insects, in an occupational therapy room. A tree at the hospital appeared to be a young woman.

**Figure 2:**
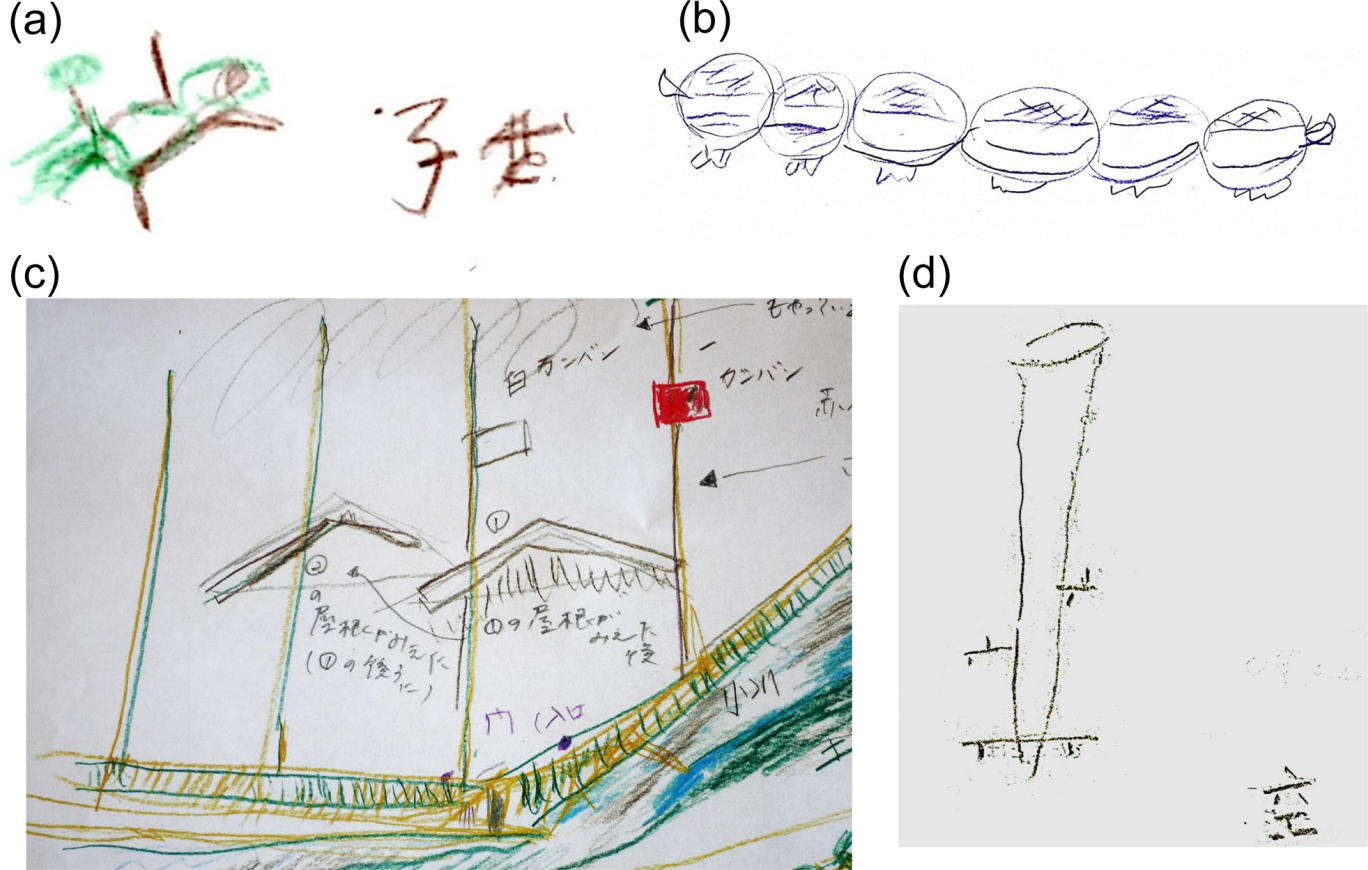
A selection of the patients’ drawings of visual illusions. (a) Polyopia (two images)/cerebral diplopia. The patient reported: ‘Just the upper body of a child at a nearby playground appeared to double’. On the right of the drawing, she wrote the Japanese characters for ‘child’ (子供). (b) Polyopia (≥three images). A single cup on the table appeared as six cups. (c) Polyopia (two images)/cerebral diplopia and polyopia (≥three images). ‘There was just one building on a playground in the neighbourhood; after looking at its roof, I could see one more roof behind it’ (polyopia (two images)/cerebral diplopia). ‘I saw three more poles when there was just one’ (polyopia (≥three images)). (d) Upside‐down illusion. The patient reported, ‘A utility pole in the neighbourhood looked upside down. I could see the sky underneath it too’. On the lower right of the drawing, she wrote the Japanese character for ‘sky’ (空).

Figure 2 presents some examples of patient drawings of experiences classified as polyopia (two images)/cerebral diplopia, polyopia (≥three images), and upside‐down illusions.

One patient reported a concerning experience not included in the illusions identified in the questionnaire. Specifically, the complaint was ‘Sometimes the ground, hospital hallway, and rehabilitation room floor look like they are going downhill; therefore, I get scared and cannot walk anymore’. This was attributed to a novel illusion, where a surface's orientation appears different from that in reality.

Responses were obtained from 26 of the 30 patients who reported any illusion(s) regarding whether they still occurred, as well as the frequency of occurrence; among them, only one patient reported having stopped experiencing illusions. Regarding frequency, 5, 10, and 11 respondents reported seeing the illusion(s) 1–5 times per day, 0.5–3 times per week, and 0.5–3 times per month, respectively. Affirmative responses were obtained from 21 patients regarding the duration of illusion occurrence; specifically, 9, 10, and 2 patients reported durations of 1–2 months, 0.3–3 years, and 5–7 years, respectively.

Regarding the impact of the visual illusions, the number of PD patients who reported that visual illusions caused problems in their activity of daily living was 12. Examples of the responses to questions —i.e. whether they experienced difficulties as a result—are provided below.

**Table:** 

Dysmorphopsia:	‘I get worried when the bed legs look bent to me, but not to others. I start arguing with the nurse about those legs’. ‘The room's door sometimes looks distorted and slanted open from the top. I cannot shrink away, and it is difficult to touch it and check when I cannot move my body well’.
Macropsia:	‘When just the handle of my mug appears bigger, my hand misses it when I go to pick it up’.
Teleopsia:	‘When I sit on my bed, I have to deliberately feel around to check the distance from me’.
Complex visual illusions:	‘When the table pattern looks like insects, it makes me want to use a different table: I get grossed out and cannot undergo rehabilitation’.

Additionally, one patient said ‘when I experience false perception of surface orientation, I just feel like, “This again?”, but it bothers me when the floor starts looking like downhill during rehabilitation’. Medical staff had noticed that the patient would occasionally stop walking and come to a standstill; however, it was attributed to freezing of gait.

The questionnaire had excellent test–retest reliability (κ = 1.0); specifically, the patients’ first‐ and second‐round responses were in complete agreement for all items.

### Visual hallucinations

Twenty‐one of the 30 patients with visual illusions had experienced visual hallucinations, and of the 10 patients without visual illusions, only one had visual hallucinations.

## DISCUSSION

In total, 75% of the included patients reported having experienced some type of visual illusion, which suggests that the prevalence of visual illusions in PD may be quite high.

In 2000, Fénelon *et al*. found that presence hallucinations (or feeling of presence), passage hallucinations, and ‘visual illusions’ occurred frequently in patients with PD and called them ‘minor hallucinations’.^23^ The ‘visual illusions’ they reported were the ‘transformation of an object into an animal (e.g. a branch was seen as a cat for a few seconds)’; hence, ‘visual illusions’ in their report was perhaps limited to complex visual illusions. Since then, a number of studies have been reported regarding minor hallucinations, but only a few reports have focused on visual illusions. In the latter studies, the relationships between visual illusions and excessive daytime somnolence and disease severity’,^24^ visual illusions and REM sleep behaviour disorder,^25^ and visual illusions and ocular disease such as epiphora and ocular anatomy impairments,^26^ have been reported. These reports also mentioned that the factors related to visual illusions may differ from those of visual hallucinations. In these studies, the questions asked for assessing the presence of visual illusionary experience were framed about complex visual illusionary experiences, since the examiner asked the patients if they had the experience of perceiving something different from what it was (e.g., animals). Conversely, only three previous studies had taken up the topic of non‐complex visual illusions. The types of the visual illusions taken up were kinetopsia, dysmorphopsia, metachromatopsia, macro‐/micropsia, tele‐/pelopsia,^2^ selective diplopia,^3^ and tilt illusions.^4^

In the current study, compared with the group without illusions, the group with at least one illusion had a longer disease duration, as well as poorer motor function, general cognitive function, long‐term memory, and visual perception. These deficits could be attributable to background factors responsible for—or sharing a common cause with—the appearance of visual illusions. However, patients with PD normally experience a decline in these functions with disease progression. Therefore, the between‐group differences could merely reflect a parallel trend of an increased illusion prevalence associated with disease progression. Noise pareidolia was common among patients who reported visual illusions. Noise pareidolia is very similar to certain complex visual illusions, including the perception of wrinkles in sheets as a human face reported by a patient. This is indicative of a close relationship between pareidolia and visual illusions. However, since the same argument as above still holds (i.e. noise pareidolia susceptibility may increase as the disease progresses, independently of visual illusion susceptibility), it is impossible to definitively draw this conclusion based solely on our results. Contrastingly, there was no between‐group difference in the LED or colour vision. This finding is interesting since dopaminergic activation and colour deficits, which are often seen in PD, may not relate to visual illusions.

The presence of varying illusions was determined based on the patients’ reports. This study identified previously reported illusions, including kinetopsia, dysmorphopsia, metachromatopsia, macropsia, micropsia, teleopsia, pelopsia, polyopia (two images)/cerebral diplopia, and tilt illusion. Tilt illusion has been reported in a case study of a single patient^4^; however, its prevalence in our study was 3/40 patients, which suggests that this illusion type is not as rare as previously thought. Moreover, this study reported several illusion types previously reported only in cases of localised brain injury, epileptic seizures, or migraine. These include akinetopsia, the Zeitraffer and Zeitlupen phenomena, upside‐down illusion, polyopia (≥ three images), and palinopsia. Additionally, one patient reported falsely perceiving the surface orientation (inclination), which had not been specifically targeted in the questionnaire. Over the last century, there have been reports of pathologies affecting general depth perception, including planar tilt.^27^ To our knowledge, there has been no report of the *selective* pathology of the ability to judge surface orientation in cases of PD, localised brain injury, epileptic seizures, or migraine headache. The perception of this illusion may be associated with dysfunction of the intraparietal sulcus, which has been shown to selectively react to surface orientation in primate physiological experiments and human functional MRI studies.^28^

Most of the patients who answered that they had an experience of visual illusions reported that the illusions persisted, with the frequency ranging from a minimum of once every 2 months to a maximum of several times every day. This suggests that many patients with PD experience illusions with considerable frequency. The patients claimed that their illusions affected their lives in several ways, including worry, discomfort, arguments with other individuals, misjudgements when grasping, the need for tactile confirmation, and stopping while walking. Furthermore, their narratives indicated some potentially dangerous illusions, including signboards and other surroundings appearing closer than in reality while driving, as well as a moving car on the highway appearing to stop suddenly. These findings indicate that systematic patient interviews regarding the incidence and details of visual illusions can offer important information about the quality of life and risk management in patients with PD.

Regarding the relationship between the presence/absence of visual illusions and that of visual hallucinations, there were more patients with visual illusions than those with visual hallucinations, and the visual illusionary experience group included the visual hallucinatory experience group except for one patient. This result may suggest that the conventional idea that minor hallucinatory phenomena, including complex visual illusions, seem to antedate the development of visual hallucinations,^29^ holds true even if the range of the visual illusions is expanded to various simple visual illusions.

Based on previous studies on patients with localised brain injuries and epilepsy, the sites of brain lesions responsible for some of the observed illusions can be surmised to a certain extent. For example, the temporo‐parieto‐occipital junction, superior parietal lobule, and intraparietal sulcus have been implicated in kinetopsia.^14^, ^15^ Further, the angular gyrus, occipitotemporal cortex, and secondary visual cortex have been implicated in the Zeitraffer phenomenon,^18^ micropsia,^12^ and cerebral diplopia,^30^ respectively. Functional MRI and other studies have demonstrated that the medial occipitotemporal cortex is critical for the perception and recognition of colour and texture.^31^, ^32^ Additionally, as previously mentioned, regions necessary for perceiving surface orientation are located within the intraparietal sulcus.^28^ All of the aforementioned brain regions have been conjectured as foci of aberrant function in PD.^33^ Therefore, dysfunction in these areas may be involved in developing visual illusions in patients with PD. However, the present study cannot clarify why only certain types of visual illusions occur in certain patients. In the future, it may be possible to shed light on this by conducting functional MRI studies or determining the characteristics of cerebral glucose metabolism using [18F] fluorodeoxyglucose positron emission tomography, in patients with PD with visual illusions.

This study has several limitations. First, we may not have identified all possible visual illusions in PD because of the small number of patients. Second, this was a single‐centre study with a small population size, which indicates that our prevalence estimates—both for illusions generally and specific illusion types—may be inaccurate. Third, although we checked the test–retest reliability of our questionnaire instrument, not all participants were re‐interviewed. Therefore, the risk of some responses being confabulations or other errors not grounded in real experiences cannot be completely discounted. Fourth, as there are many types of confirmed visual illusions and the strength of the relationships among each illusion could vary, it would be difficult to analyse the relationship between the background and illusion because of the small number of participants. This may be clarified by increasing the number of participants, performing factor analysis on illusions which the participants are experiencing at the time of the survey, and analysing the correlation between the extracted illusion groups and backgrounds. Fifth, in the current study, patients with NVA < 0.5 were excluded because it could possibly affect the results of the Visual Perception Test for Agnosia and Noise Pareidolia Tests. NVA in PD patients has been reported to be worse than in healthy individuals.^34^ In addition, Marques *et al*. reported that patients with PD with complex visual illusions had more epiphora and ocular anatomy impairments than healthy subjects.^26^ There was no significant difference in visual acuity between patients with PD and healthy subjects in this study. However, to the best of our knowledge, there are no studies examining the relationship between each simple visual illusion and NVA. Therefore, it is possible that NVA impairment can lead to simple visual illusions. From this point of view, the 75% prevalence of visual illusions in the current study may actually be higher. Additionally, patients with cognitive impairment were not excluded in the current study. However, of the 30 patients with PD with visual illusions, only three scored below 21 in the MoCA‐J, and were considered as having PD with dementia.^35^ Therefore, it is unlikely that the presence of dementia has influenced the results of the current study. However, the MoCA‐J score of the patients in the visual illusion group was significantly lower than that of the patients without visual illusions. Therefore, it cannot be ruled out that milder general cognitive impairment may affect the answers to the questions.

## CONCLUSION

This study showed that most of the surveyed patients with PD reported many types of visual illusions, with some being different from those previously documented in the PD literature. Some illusions interfered with the patients’ daily lives. Systematic patient interviews regarding the incidence and details of visual illusions could offer important information about the quality of life and risk management in patients with PD.
